# Impairment in Emotional Intelligence May Be Mood-Dependent in Bipolar I and Bipolar II Disorders

**DOI:** 10.3389/fpsyt.2021.597461

**Published:** 2021-02-18

**Authors:** Shih-Yu Kuo, Yun-Hsuan Chang, Tzu-Yun Wang, Huai-Hsuan Tseng, Chih-Chun Huang, Po See Chen, Hsien-Yuan Lane, Yen Kuang Yang, Ru-Band Lu

**Affiliations:** ^1^Department of Psychology, Asia University, Taichung, Taiwan; ^2^Clinical Psychological Center, Asia University Hospital, Taichung, Taiwan; ^3^Department of Medical Research, China Medical University Hospital, Taichung, Taiwan; ^4^Department of Psychiatry, National Cheng Kung University Hospital, College of Medicine, Tainan, Taiwan; ^5^Institute of Behavioral Medicine, College of Medicine, National Cheng Kung University, Tainan, Taiwan; ^6^Department of Psychiatry, National Cheng Kung University Hospital, Dou-Liou Branch, Yunlin, Taiwan; ^7^Department of Psychiatry and Brain Disease Research Center, China Medical University Hospital, Taichung, Taiwan; ^8^Graduate Institute of Biomedical Sciences, China Medical University, Taichung, Taiwan; ^9^Department of Psychiatry, Tainan Hospital, Ministry of Health and Welfare, Tainan, Taiwan; ^10^Yanjiao Furen Hospital, Hebei, China

**Keywords:** emotional intelligence, duration of illness, euthymia, mood episodes, bipolar disorders

## Abstract

**Background:** An emotional intelligence (EI) deficit has been noticed in euthymic bipolar spectrum disorder (BD) patients. However, whether this deficit is affected by mood or subtype is unclear.

**Objectives:**The aim of this study was to investigate whether an EI deficit is mood-dependent, and which mood symptoms have more impact on EI in BD.

**Methods:** Two hundred and thirty participants aged between 18 and 65 years old were recruited [130 BD patients (51 bipolar I disorder (BDI) and 79 bipolar II disorder (BDII): 39.2% males; 91 healthy controls (HCs): 48.4% males)]. The Mayer–Salovey–Caruso Emotional Intelligence Test (MSCEIT), which contains experiential and strategic EI ratings, was used to assess social cognition. The Hamilton Depression Rating Scale (HDRS) and the Young's Mania Rating Scale (YMRS) were used for evaluating the severity [HAMD and YMRS scores ≦7 were euthymic (BD^eut^) and HAMD YMRS sores ≧8 were episodic (BD^epi^)]. Analyses of covariance (ANCOVA) were performed, with adjustment for background information between the BD patients and HCs.

**Results:** The results showed that, compared to the HCs, the BD^eut^ patients showed no difference in any MSCEIT measures, while the BD^epi^ patients showed lower scores in all MSCEIT measures, except for perceiving emotions. In addition, a main effect of mood state instead of BD subtype was found for the managing emotions branch (*p* < 0.0007). Regression analyses showed that the duration of illness and HDRS scores were correlated with the scores in the strategic area of the MSCEIT, while age and YMRS scores were more relevant to the scores in the experiential area of the MSCEIT.

**Conclusion:** The results confirm that an EI deficit is mood-dependent in BD patients. In addition, a depressive mood is more related to the strategic EI area, while a manic mood is correlated with the experiential EI area. Understanding the different domains of EI deficits in BD patients may be helpful for developing interventions for BD.

## Highlights

- No significant differences of poor emotional intelligence between subtypes of bipolar spectrum disorders.
- Impairment in managing emotions could be considered a psychopathological factor in BD.
- Depressive and manic moods have different impacts on different facet of ability emotional intelligence in patients with BD.

## Introduction

Bipolar spectrum disorder (BD) is recognized as a severe and chronic mental disorder (1, 2). A similar lifetime prevalence of BD has been reported among genders and ethnicities (3). Due to the high relapse rate and mixed mood swings, life dysfunction, such as sleep disturbance, difficulty in maintaining interpersonal relationships, and poor performance at work, is common in BD. About 60% of BD patients have poor social interpersonal relationships (4), and it appears that there is an association between mood episodes and social functioning (5, 6). However, the exact nature of this association has not been determined to date and requires further investigation (7).

BD has been recognized as a mental disorder with emotional dysregulation (8). Previous studies reported social cognitive impairment in patients with BD during their remitted states (9, 10). Studies have compared the ability to identify and recognize the facial expressions between BD patients and controls; however, the results have been inconsistent (11, 12). BD patients often suffer from emotional disturbance related to skills regarding social information, emotional processing, and emotional regulation, and this has been suggested as one of the core symptoms in BD (7, 8).

Gruber et al. (13) suggested that emotional dysregulation could be the core deficit in BD, and thus, patients with BD have difficulty regulating their emotions and lack a higher level of social cognition. BD patients may have similar performance in regulating their emotions but fail in the engagement of maintaining this regulation performance compared with healthy controls and need more cues and coping skills (13). A small-to-moderate difference in the social cognitive performance between euthymic BD patients and healthy controls (HCs) has been reported, indicating that mental state decoding is more preserved than higher levels of social cognition and mental state reasoning (14).

Social cognition refers to the ability to identify, perceive, and interpret socially relevant information (15–18), to construct differences between others and oneself (19). Five domains of social cognition have been suggested by the National Institute of Mental Health (NIMH), namely, theory of mind, social perception, social knowledge, attribution bias, and emotional processing (20). However, these domains are still under debate (7). Emotional processing includes recognizing facial expressions and evaluating others' emotional states (18). Some researchers have argued that emotional processing includes low-level cognitive processing, such as emotional awareness and distinguishing facial expressions. A high level of social cognition is involved in emotional management and emotional regulation, which are more complicated (21–23).

The ability to identify and interpret others' facial expressions and information is important for social communication and interpersonal relationships (24). Salovey and Mayer (25) constructed the emotional intelligence (EI) theory and developed the Mayer–Salovey–Caruso Emotional Intelligence Test (MSCEIT) (26) as a measure for this element of social cognition. Mayer et al. (27) defined EI as containing a hierarchical cluster of emotion-related skills, including both the experiential and strategic areas of EI. Under each area, there are two branches, and each branch contains two tasks. For experiential EI, the ability to perceive and use emotions was constructed through the identification and recognition of facial expressions and pictures, as well as the ability to facilitate emotional information and sensation. For strategic EI, the abilities of understanding and managing emotions are included.

Referring to the social cognitive domains suggested by the NIMH, the MSCEIT contains emotional processing ability, represented by the recognition of facial expressions, social perception, and emotional processing. The MSCEIT may be used to assess EI with lower-level emotional processing, such as the recognition of facial expressions, as well as higher-level emotional regulation. Mayer et al. (28) chose the most frequent response as the correct answer in exploring whether an emotion was universal for all. Mayer and Geher (29) found an association between emotional processing and empathy. They further suggested that emotional intelligence may be required for some forms of problem solving skills.

EI has been further noted as lacking in people with severe mental disorders, such as schizophrenia (30). EI has been suggested to be a cognitive capability that plays an important role in processing emotional information (31, 32). In addition, EI, measured by the MSCEIT, has been assessed in remitted BD patients, but the results thus far have been inconsistent (33–35). A deficit in EI in remitted BD patients has been reported in certain studies (36, 37); however, only one sub-facet of the MSCEIT—the managing emotions branch—has been studied and compared. These reports imply a higher level of emotional dysregulation in remitted BD patients. However, this difference was not found between euthymic and symptomatic bipolar I disorder (BDI) individuals. Thus, researchers suggested that the impairment in managing emotions could be an endo-phenotype within BDI (10).

There are four branches of MSCEIT, and only the facet of managing emotion was tested previously (36, 37), the performance in other branches is still unclear. Researchers examined the construct of MSCEIT using meta-analysis (38), and suggested a three-factor model, combining branch 1 and 2 as one factor. In addition, the three-factor model was further tested with age invariance (39). Due to the high correlation between the first two branches—perceiving emotion and using emotion—the three-factor model—experiential EI, understanding emotions, and managing emotions—was preferred (38, 39).

Although the MSCEIT was suggested as a test of cognition-based performance in adults of all ages (40, 41), not all tasks had age invariance; for example, the sensation task of the facilitating emotion branch is more age-related; older adults showed higher mean scores in the sensation task (39). Only one facet—managing emotion—was included as cognition-related, other branches of the MSCEIT were not examined. Thus, the managing emotion branch may not present the core impairment of ability regarding emotional intelligence in BD; other branches may have variant degrees of impairment in the BD, and should be tested.

BDI has been the most focused on and studied for BD; fewer studies have focused on social cognition in bipolar II disorder (BDII), and the results have been inconclusive. Studies have reported worse facial emotion recognition in BDI patients (2, 42, 43), and others have reported similar theory of mind and emotional perception abilities between BDI and BDII patients (2, 40, 41). In addition, Van Rheenen et al. (44) reported a mediator of depressive symptomatology on emotional regulation and psycho-social functioning in BDI, indicating that depressive symptomatology played a greater impact on emotional regulation and psychological functioning in BDI. Different domains of social cognition may have different degrees of impairment related to mood episodes (42) depending on the BD subtype.

The contradictory findings of previous studies imply that the mixed mood swings often seen in BD patients may impact EI differently. Worse interpersonal relationships and poorer social adaptation than in HCs have been noted, possibly caused by an impairment in social cognition (5, 6). Approximately 50% of remitted BD patients have been reported to have impairments in this area (45), and ~55% have at least one type of social cognition deficit, even in remission (46). Poor social adaptation has been suggested to be a predictive prognostic factor (47–49). The MSCEIT was constructed in line with these abilities and has been suggested to be a reliable measurement of social cognition.

A previous study also reported that remitted BD patients have significantly lower scores in both the total scores and the branches of experiential EI and strategic EI, compared to HCs (33, 50). Remitted BD patients still demonstrate poorer EI, and, for the Han Chinese BD population, the EI aspect of understanding emotions was found to be impaired in euthymic BD patients, both in terms of BDI and BDII (50). However, the correlation with mood symptomatology is not clear. Although neuropsychological impairment has been suggested as a feature of BD, some domains of neuropsychological performance were found to be related to mood episodes (51). Thus, in the current study, we investigated whether impairment in the different sub-facets of the MSCEIT is related to BD patients' mood episodes. In addition, whether patients with BDI or BDII have different domains of EI impairment was studied. We investigated whether mood symptoms have different roles in affecting the different sub-domains of EI.

## Materials and Methods

This study received approval from the Institute Review Board (IRB) of the National Cheng Kung University Hospital (IRB#A-BR-103-078, IRB#A-BR-104-089 and IRB#A-BR-108-001) and the China Medical University Hospital (IRB# CMUH106-REC3-023 and IRB#CMUH108-REC3-024).

### Participants

Participants with a diagnosis of BD (51 BDI and 79 BDII patients), aged 18–65 years, were recruited from inpatient and outpatient clinics at two medical centers. The patients were referred from senior psychiatrists who made the initial diagnosis. All participants underwent a structured interview using the Schedule for Affective and Schizophrenia—Lifetime Chinese version (SAD-L) to confirm the diagnosis based on the Diagnostic and Statistical Manual of Mental Disorders 5th edition (DSM-5) and to screen for healthy controls. Patients with substance use disorders or major neurological disorders were excluded.

The participants' symptoms and symptom severity were evaluated using the Young Mania Rating Scale (YMRS) (52) and the Hamilton Depression Rating Scale (HDRS) (53, 54). The healthy controls (HCs) were recruited via post and an online advertisement. All of the HC participants underwent a structured interview to confirm their health condition. Those with current major mental disorders, or a history of mental disorders or neurological disease, or these disorders within their first-degree relatives were excluded.

### Assessments

The Young Mania Rating Scale (YMRS) is commonly used for the evaluation of the severity of mania (52). A YMRS score below 7 is defined as normal, 8–13 as marginal, 14–20 as mild, 21–26 as moderate, and higher than 38 as severe. For evaluation of the severity of depression, the Hamilton Depression Rating Scale_−17_ (HDRS_−17_) was used. A score of 0–3 is defined as normal, 4–7 as marginal, 8–15 as mild, 16–26 as moderate, and higher than 27 as severe (55). Based on previous studies, BD patients with HAMD and YMRS scores of ≦7 were considered euthymic (6, 34, 50, 55).

### Emotional Intelligence

To study social cognition performance, the Mayer–Salovey–Caruso Emotional Intelligence Test (MSCEIT) (26) was used because it examines several classes of social cognition, including emotional perception, emotional use, emotional understanding, and emotional management (25). Four branches are included, and each branch contains two tasks: (1) emotional perception, which includes facial expression identification and picture perception; (2) emotional use, which includes facilitation and sensation, in which the ability to use emotions to enhance and facilitate cognitive processing and to assess the correlation between emotions and sensation are measured; (3) emotional understanding, which includes change and blends, that is, to study the individual's knowledge of how emotions interact with each other and change over time; (4) emotional management, which includes the management of emotions and emotional relationships, in which how an individual regulates their emotions themselves and also with others is measured.

A total of 141 items are included in the MSCEIT. In general, the MSCEIT score reflects the general EI level, with scores from two areas: experiential EI and strategic EI. We used all of these scores, obtained using a consensus scoring criterion (22). A higher score represents better social cognition. The reliability and validity of the traditional Chinese version of MSCEIT have been established already (26, 56). The reliability in the current study showed Cronbach's α was 0.82. The reliability scores for the four branches, perceiving, using, understanding, and managing emotions were 0.91, 0.62, 0.64, and 0.74, respectively. The split half reliability scores for each branch were 0.71, 0.67, 0.68, and 0.79, respectively.

### Statistical Methods

The BD and HC groups were compared using the chi-square test or one-way analysis of variance (ANOVA), depending on the variable type. As there were significant differences in the background information, level of education, and age, an analysis of covariate (ANCOVA) was further conducted to compare the MSCEIT measures between the BD and HC groups. The Games–Howell *post-hoc* test was used for pairwise comparisons. To investigate whether the mood episodes or the subtypes were more correlated to the MSCEIT measures, multivariate analyses of variance were conducted. To compare the effect of HDRS and YMRS on the MSCEIT measures in BD, Pearson's *r* association analysis was conducted, and stepwise regression analyses were performed to investigate the effect of the clinical variables and mood symptoms on the EI performance. The statistical significance was set at *p* < 0.05. All analyses were performed with the Statistical Package for Social Sciences version 22 (SPSS Inc., Chicago, IL, USA).

## Results

### BD Patients vs. HCs

All patient groups were undergoing regular treatment. One hundred and thirty BD patients (mean age 36.98; 51 males) and 91 HCs (mean age 31.16; 44 males) were recruited. The results showed significantly older age and lower educational level for the BD group compared to the HC group (χ^2^ = 12.70, *p* < 0.0005*;* F = 33.78, *p* < 0.0005, respectively) (Table 1). In addition, the BD group had significantly lower scores in the three branches of the MSCEIT than did the HC group, except for the perceiving emotions branch of the MSCEIT. The BD group was split into episodic and euthymic groups, based on the observation of their current mood episodes (HDRS and YMRS scores of ≦7 were defined as a euthymic state (BD^eut^) and HDRS and YMRS scores of ≧8 were defined as a mood episodic state (BD^epi^).

**Table 1:** Clinical characteristics and demographic data among groups Clinical characteristics and demographic data among groups.

	**BD Groups**		**HC (*n* = 91)**	**F/χ^2^ (*p)***
	**^**1**^BD^**epi**^ (*n* = 73)**	**^**2**^BD^**eut**^ (*n* = 57)**		
Age	35.58 (11.94)	38.79 (13.84)	31.16 (10.51)	7.55 (0.001)
Educational level	13.34 (3.85)	12.95 (4.68)	15.79 (2.06)	17.03 (< 0.0005)
Gender (Male, %)			44 (48.4%)	
Onset	16.37 (6.54)	16.39 (6.68)	–	–
Duration of illness	17.59 (11.30)	20.33 (14.18)	–	0.63 (0.43)
HDRS	10.49 (3.61)	2.84 (3.03)	–	187.69 (<0.0005)
YMRS	9.70 (3.67)	2.96 (3.03)	–	125.24(<0.0005)
Comorbidity rate (%)	33.3%	7.4%	–	13.76 (<0.0005)
No. of Comorbidity (%)			–	11.87 (0.003)
1	19.0%	5.6%		
2	14.3%	1.9%		
Treatment (%)			11.82	0.02
Mood stabilizers (VPA or lithium)	22.6%	4.1%	–	–
VPA + antipsychotics	25.8%	44.9%	–	–
Antipsychotics	1.6%	6.1%	–	–
BZD	1.6%	0		
Other medications (SSRI or no medication use)	22.6%	4.1%	–	–

*VPA, valproic acid; 1, Episodic BD group; 2, Euthymic BD group*.

**Figure 1:**
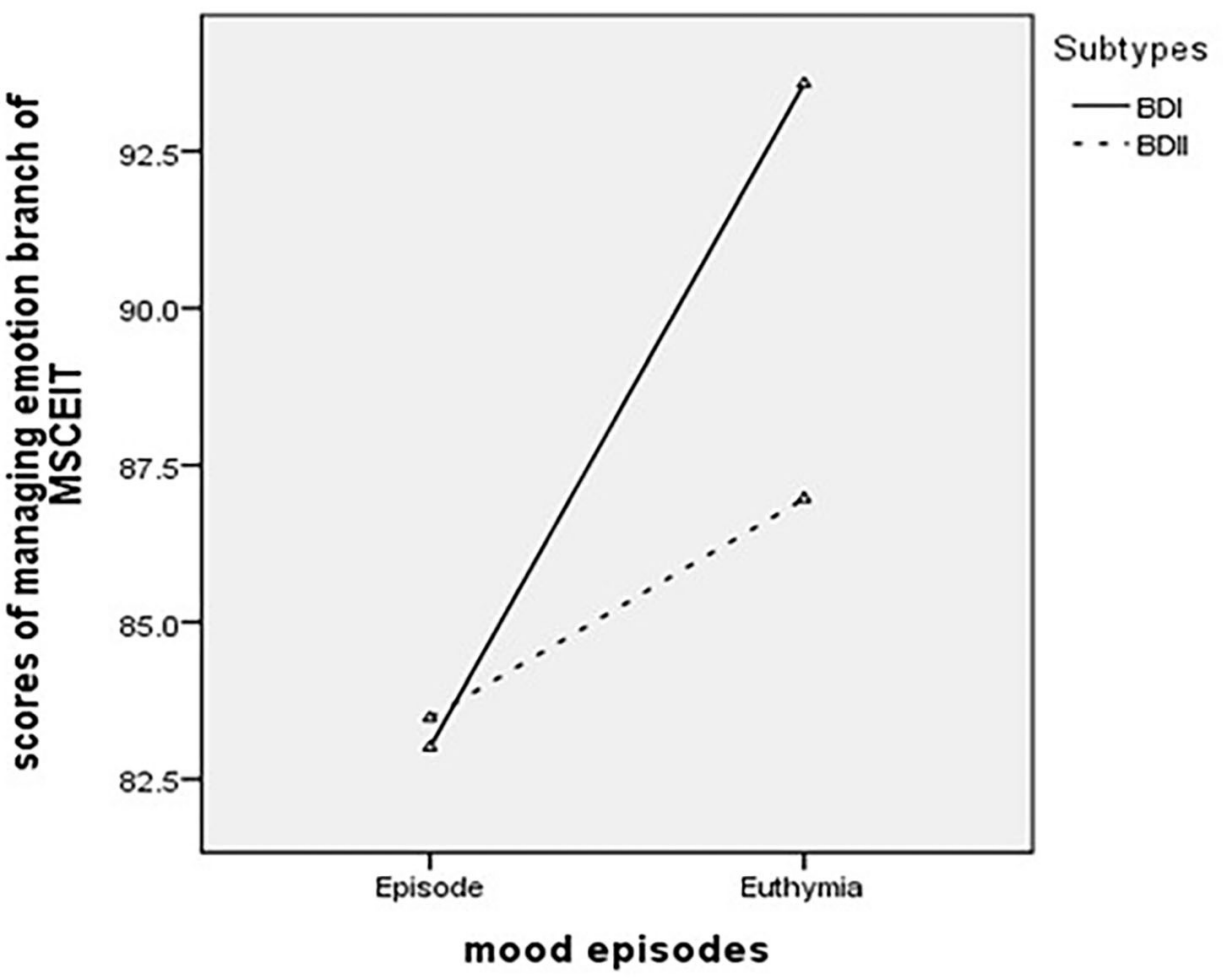
The main effect of mood state in scores of managing emotion branch of MSCEIT.

Multivariate analysis of the covariate analyses revealed a significant mood episode effect on the EI area scores of the MSCEIT (general linear model, Wilk's Lambda = 0.93, *p* = 0.036). Additionally, a significant main effect of mood episodes on the branches of the MSCEIT as found (*F* = 2.70, Wilk's Lambda = 0.92, *p* = 0.03). After Bonferroni correction (0.05/4 = 0.0125), the managing emotions branch of strategic EI remained significantly different between episodes and the euthymic state (*p* < 0.0007) (Figure 1).

### Correlation Between EI and Mood Episodes

To investigate whether EI impairment was affected by mood episodes, 57 BD^eut^ patients and 73 BD^epi^ patients were tested. The BD^epi^ group had significantly lower total scores, in both EI areas and the four branches of the MSCEIT compared with the HC group. The BD^eut^ group had similar scores in all MSCEIT branches compared with the HC group, except in the understanding emotions branch of the MSCEIT (*p*< *0.004*). No significant differences in the scores for the understanding emotions branch of the MSCEIT were found between the BD^epi^ and the BD^eut^ groups. However, the BD^epi^ group was found to have significantly lower scores in managing emotions on the MSCEIT compared with the BD^eut^ group (Table 2).

**Table 2:** Comparisons between BD groups and controls with different mood states with respects to emotional intelligence.

	**BD groups**		**^**3**^HC (*n* = 91)**	**Analysis of variance**				**Analysis of covariance controlling for age & education level**			
				***F***	**1 vs.2**	**1 vs.3**	**2 vs. 3**	***F***	**1 vs.2**	**1 vs.3**	**2 vs. 3**
	**^**1**^BD^**epi**^ (*n* = 73)**	**^**2**^BD^**eut**^ (*n* = 57)**									
MSCEIT^a^											
Total score	82.59(16.84)	90.09(18.40)	94.67(12.86)	11.92^**^	0.02	<0.0005	n.s	14.43^**^	0.006	0.001	n.s
*Area1: Experiential EI*	91.61(21.18)	100.38(21.55)	103.34(16.52)	7.61^**^	n.s	<0.0005	n.s	5.87^**^	0.04	0.03	n.s
Branch 1: Perceiving Emotions	95.09(19.05)	103.65(22.14)	103.46(18.88)	4.44^*^	n.s	0.02	n.s	3.86^**^	n.s	n.s	n.s
Branch 2: Using Emotions	88.82(20.36)	95.82(20.30)	101.53(15.15)	9.69^**^	n.s	<0.0005	n.s	6.76^**^	n.s	0.003	n.s
*Area 2: Strategic EI*	80.57(11.55)	84.28(13.43)	88.95(9.97)	10.96^**^	n.s	<0.0005	n.s	26.52^**^	0.02	0.004	n.s
Branch 3: Understanding Emotions	81.72(13.50)	82.12(12.59)	88.63(9.30)	8.89^**^	n.s	0.001	0.003	35.78^**^	n.s	0.04	n.s
Branch 4: Managing Emotions	83.33(12.23)	90.33(15.82)	92.27(12.99)	9.30^**^	0.019	<0.0005	n.s	9.24^**^	0.008	0.01	n.s

*Data represented as Mean (SD)*;

a *MSCEIT, Mayer–Salovey–Caruso Emotional Intelligence Test*;

* *p < 0.05*;

** * < 0.007 (Bonferroni correction for multiple comparisons); 1, episodic BD group; 2, euthymic BD group; 3, HC group*.

### Correlation Between Mood Symptoms and EI

Correlation analysis showed a significantly negative association between the HDRS, the YMRS, and the total scores of the MSCEIT. The results showed a negative association between the HDRS, the YMRS, and the scores in the experiential area of the MSCEIT. The only significantly negative association was found between the HDRS scores and the scores in the strategic area of the MSCEIT. A significantly negative association was found between the HDRS, the YMRS, and the scores on the three MSCEIT branches, except for the scores of the understanding emotions branch of the MSCEIT (Table 3).

**Table 3:** Correlation matrix between clinical characteristic, mood severity and MSCEIT scores.

	**1**	**2**	**3**	**4**	**5**	**6**	**7**	**8**	**9**	**10**	**11**	**12**	**13**	**14**	**15**	**16**	**17**	**18**	**19**	**20**
1.Age	1	0.31^*^	0.86^***^	−0.08	−0.14	−0.37^***^	−0.22^*^	−0.18^*^	−0.15	−0.21^*^	−0.24^**^	−0.08	−0.37^***^	−0.54^***^	−0.65^***^	−0.51^***^	−0.63^***^	−0.27^**^	−0.33^***^	−0.17
2.Onset		1	−0.23	0.05	−0.4^**^	0.06	0.16	0.19	0.19	0.11	0.15	0.20	0.12	−0.16	−0.22	−0.21	−0.19	−0.06	0.06	−0.12
3.Duration of illness			1	−0.06	0.17	−0.35^*^	−0.20	−0.13	−0.02	−0.23	−0.31^*^	−0.13	−0.46^***^	−0.49^***^	−0.54^***^	−0.34^*^	−0.56^***^	−0.31^*^	−0.37^*^	−0.22
4.HDRS				1	0.50^***^	−0.26^***^	−0.24^*^	−0.19^*^	−0.14	−0.23^**^	−0.20^*^	−0.27^**^	−0.03	−0.20^*^	−0.04	−0.07	0.01	−0.31^***^	−0.28^***^	−0.29^***^
5.YMRs					1	−0.25^**^	−0.28^***^	−0.23^*^	−0.19^*^	−0.27^**^	−0.27^**^	−0.34^***^	−0.12	−0.01	0.02	0.004	−0.13	−0.23^**^	−0.21^*^	−0.21^*^
6. Total score						1	0.94^***^	0.89^***^	0.65^***^	0.71^***^	0.88^***^	0.74^***^	0.77^***^	0.89^***^	0.70^***^	0.62^***^	0.56^***^	0.81^***^	0.74^***^	0.71^***^
7. Exp. EI							1	0.78^***^	0.71^***^	0.77^***^	0.90^***^	0.80^***^	0.75^***^	0.70^***^	0.54^***^	0.49^***^	0.41^***^	0.65^***^	0.59^***^	0.56^***^
8. Branch 1:Perceiving emotions								1	0.77^***^	0.86^***^	0.52^***^	0.45^***^	0.48^***^	0.45^***^	0.37^***^	0.35^***^	0.27^**^	0.40^***^	0.34^***^	0.35^***^
9.Face									1	0.55^***^	0.53^***^	0.46^***^	0.49^***^	0.45^***^	0.39^***^	0.33^***^	0.30^***^	0.37^***^	0.31^***^	0.36^***^
10.Picture										1	0.54^***^	0.48^***^	0.52^***^	0.51^***^	0.42^***^	0.42^***^	0.31^***^	0.45^***^	0.39^***^	0.41^***^
11.Branch 2: Using emotions											1	0.89^***^	0.81^***^	0.69^***^	0.53^***^	0.46^***^	0.43^***^	0.65^***^	0.60^***^	0.56^***^
12. Facilitation												1	0.50^***^	0.52^***^	0.34^***^	0.29^***^	0.25^**^	0.55^***^	0.51^***^	0.49^***^
13. Sensation													1	0.68^***^	0.59^***^	0.53^***^	0.50^***^	0.5^***^	0.52^***^	0.48^***^
14.Strategic EI														1	0.82^***^	0.72^***^	0.71^***^	0.85^***^	0.79^***^	0.76^***^
15. Branch 3: Understanding emotions															1	0.86^***^	0.87^***^	0.43^***^	0.41^***^	0.35^***^
16. Changes																1	0.52^***^	0.38^***^	0.35^***^	0.32^***^
17. Blends																	1	0.35^***^	0.36^***^	0.28^**^
18. Branch 4: Management of emotions																		1	0.89^***^	0.92^***^
19. Emotional management																			1	0.65^***^
20. Emotional relationships																				1

* *p <0.05*;

** *p < 0.01*;

*** *p < 0.001*.

Subsequently, regression analyses showed that the duration of illness and HDRS score was predictable for the total score of the MSCEIT. The age was predictive to both EI areas, experiential and strategic. In addition, different roles of the HDRS and YMRS were found correlated to different EI areas of MSCEIT; the YMRS was more related to the scores on experiential EI, while the HDRS was more related to the strategic EI. To explore whether the depression or (hypo)mania symptomatology had different impacts on different branches, the results showed that the scores on the perceiving emotion branch were affected by the YMRS and educational level while the duration of illness affected the scores on the using emotion branch. For the branches of the strategic EI area, the results showed that age played an important role on the using emotion branch, while the HDRS was negatively correlated to the scores on the managing emotion branch of MSCEIT (Table 4).

**Table 4:** Multiple regression analysis model coefficients for emotional intelligence ability through MSCEIT branches.

**Variables**	**Model 1**			***F* (*p*)**	**Model 2**			***F* (*p*)**
	**B**	**SE_**B**_**	**β**		**B**	**SE_**B**_**	**β**	
MSCEIT^a^								
Total								
duration	−0.55	0.18	−0.37	9.38 (0.003)	−0.58	0.17	−0.40	7.43(0.001)
HDRS					−1.17	0.53	−0.26	
*Area1: Experiential EI*								
YMRS	−1.29	0.39	−0.28	11.10 (0.001)	−1.46	0.38	−0.32	11.26(<0.0005)
Age					−0.45	0.14	−0.27	
Branch 1: Perceiving Emotion								
YMRs	−0.99	0.38	−0.23	6.89 (0.01)	−1.13	0.37	−0.26	6.90 (0.001)
Educational level					−0.35	0.14	−0.22	
Branch 2: Using Emotion								
Duration	−0.53	0.22	−0.30	6.09 (0.016)				
*Area 2: Strategic EI*								
age	−0.58	0.11	−0.59	30.81(<0.0005)	−0.59	0.10	−0.60	33.96(<0.0005)
HDRS					−0.63	0.18	−0.25	
Branch 3:Understanding Emotions								
age	−0.71	0.11	−0.63	38.38(<0.0005)				
Branch 4: Managing Emotions								
HDRS	−0.83	0.24	−0.30	11.54 (0.001)	−0.093	0.24	−0.34	11.04 (<0.0005)
Educational level					0.90	0.29	0.27	

*MSCEIT, Mayer–Salovey–Caruso Emotional Intelligence Test*;

a *Raw scores of MSCEIT-TC in each branch, area, and total score*.

## Discussion

This study investigated the association between emotional intelligence and mood episodes in patients with bipolar spectrum disorder. In addition, we investigated the differences in the full scales of the MSCEIT between the BD subtypes, BDI and BDII. The correlation between mood symptomatology (depression or mania) of BD with EI sub-facets was considered. Our findings showed that, overall, BD patients suffering from a mood episode had lower scores on total score of MSCEIT, and on the experiential, strategic, and managing emotions functioning compared to those who were euthymic. In addition, mood episodes had a greater effect, compared with the subtype, on managing emotions from the strategic EI branch of the MSCEIT. Strategic EI was more correlated to depressive scores, while experiential EI was more correlated to the severity of (hypo)mania scores.

In regard to emotional intelligence, the results showed that, regardless of background information, the BD^eut^ groups had significantly lower scores on the understanding emotions branch compared to the HCs, while the BD^epi^ group had lower scores on all subscales of the MSCEIT. Our findings are similar to those of Liu et al. (50), who reported that euthymic BD was associated with relatively lower scores on the understanding emotions branch of the MSCEIT compared to HCs.

However, after adjustment of the background information, specifically the age and education level, the BD^eut^ group had no significant differences on any of the subscales of the MSCEIT compared to the HCs, while the BD^epi^ groups still had significantly lower scores in experiential EI and strategic EI as well as all branches—except for the perceiving emotions branch of the MSCEIT. The interaction between age and educational level has been suggested to be highly correlated with EI, and the educational level has been suggested to preserve EI with age decline (57). The BD patients in the current study showed older age and lower educational levels—both factors may have influenced the total EI and branches.

The impairment of the understanding emotions of the MSCEIT was noted in euthymic BD patients, which is consistent with previous findings (33, 50). In addition, regardless of mood episodes, the lower scores on the understanding emotions branch in both the BD^epi^ and BD^eut^ groups compared to the HC imply a possible endo-phenotypic symptom in BD. The scores on the managing emotions branch of the MSCEIT were found to be significantly different between BD^epi^ and BD^eut^; however, no significant difference was found between BD^eut^ and the HC.

This finding may indicate that the managing emotion ability is the most impacted subdomain of emotional intelligence in the patients suffering from BD. The performance in the managing emotions branch typically predicts a majority of mental disorders, such as schizophrenia (58) and bipolar disorder, compared to unaffected siblings (59). In the current study, we found that the difference in the managing emotions scores of the MSCEIT between the BD^epi^ and BD^eut^ groups remained significant, implying that the ability to manage emotions could be mood-dependent and a state-like marker for the evaluation of the progressive course of the illness.

In addition, the age range of the sample was huge, although we did not find an age effect as reported in previous studies (32, 60). Cabello et al. (60) reported an inverted U shape for the effect of age on emotional intelligence in normal adults, where those aged 32–44 years old showed better performance on the MSCEIT. In the current study, the mode of the duration of illness was 11 years, and the median was 16 years. The duration of illness and the residual mood symptomatology may have a greater impact than age on the using emotion branch of the MSCEIT; a larger sample could be tested in future to investigate this effect.

Our findings were in line with previous findings that the social cognition performance in BD patients is affected by mood episodes and the severity of the mood (6, 30, 61). A previous report showed that as the severity of depression increased, the degree of social cognition impairment increased in patients with major depressive disorder (30). Depressive episodes are considered as a criteria for a BD diagnosis; the course of this part of the illness may affect the diagnosis and treatment (6, 62). Exploring the association between a patient's depressive course and their manic/hypomanic course and social cognition is important in understanding the changes of social cognition in BD. A longitudinal study should be carried out to investigate whether the impairment in emotional intelligence is mood-dependent and to determine the changes that occur according to the disorder's course.

The negative association between the duration of illness and total scores of the MSCEIT implies a possible characteristic marker (26). Further studies focusing on the relationship between emotional regulation and mood symptoms could be considered (13, 63). A negative association between mood symptoms and MSCEIT measurement was noticed in the BD group, suggesting both a deficit in social cognition during mood episodes in BD patients and that the MSCEIT is reliable and can be taken into consideration when evaluating patients with mood disorders (56).

The only significant difference was in the understanding emotions branch of the MSCEIT between the BD^eut^ group and the HCs; however, this difference disappeared after adjustment for age and education. This is in agreement with previous findings that some domains of social cognition may be intact in euthymic or remitted BD patients (16, 62). For the other branch of strategic EI, managing emotions, our results showed that this impairment was more mood-dependent and state-like, as no significant difference was found between the BD^eut^ group and the HCs, but a difference was found between the BD^epi^ and BD^eut^ groups. However, the scales in the managing emotions branch for the BD group represented lower kurtosis; this may reflect that patients in this such sub-facet have impairment compared to the HC (normal distribution).

We further, separately explored the distribution between BD^epi^ and BD^eut^, and we found that in the BD^epi^, the scores in the managing emotions branch was in violation of the normal distribution. This may reflect that the BD patients suffered from their disorder course and indicate that the impairment in emotional management could be a core feature in BD. In addition, this finding indicates that this impairment may be a mood-dependent marker for therapeutic effects in BD. A longitudinal study should be carried out to confirm this effect. An association between cognitive functioning and social cognition has been suggested (37). A previous study reported that, in BD patients with intact neuropsychological functioning, a lesser impairment of managing emotions was found (64). However, in the current study, premorbid IQ was not recorded, and except for excluding those with IQ <80, the BD patients' cognition levels were not measured.

Previous studies have reported that depressive symptoms may have more of an effect than (hypo)manic symptoms on EI performance (6, 62), and a negative association between the mood severity and different areas of the MSCEIT was found in our study. This may imply that different mechanisms are active during depression and (hypo)mania in processing and regulating emotions in BD. BD patients, during a manic episode, have been reported to have greater impairment in cognitive functioning and poorer performance in emotional experiences and perceptions; a significant negative correlation between YMRS scores and age was found in our study.

In addition, the duration of illness and HDRS scores were negatively correlated with managing emotions, which may show that depressive symptoms have a greater impact on higher cognitive functions (30, 62, 63). A previous study reported that cognitive deficits were more pervasive in BDII, and suggested that depressive episodes, rather than manic episodes, may have a greater impact and a lasting effect on cognitive deficits (2). In addition, strategic EI ability was reported to be highly associated with IQ and cognition (37). The association between depressive symptoms and strategic EI scores in our study confirmed the greater impact of depression on EI.

The literature has documented that people with BD take longer to complete the MSCEIT, and we noted that the BD^epi^ group took significantly longer for MSCEIT completion, while the BD^eut^ group showed no significant difference compared to the HCs. Most of the episodic BD patients spent much more time than estimated in the handbook, which may reduce its clinical use for BD patients during mood episodes. In addition, another limitation in the current study was that medication was not fully recorded, and there was no investigation of the association between medications and emotional intelligence.

There were several limitations of the current study. The first limitation was that the IQ and cognition levels were not recorded, although participants with an IQ of ≦80 were excluded. In addition, the heterogeneity of the BD group limited the results, and the clinical course, such as the number of depressive or manic/hypomanic episodes and the number of hospitalizations, was not recorded. The effect of comorbidities was not considered when studying emotional intelligence in BD, though a high comorbidity rate in BD has been reported.

We recorded only the number of comorbidities; therefore, the comorbidity types could be recorded in further investigations. The managing emotion branch may be predictable for the majority of mental disorders and associated with general distress, while the mood course in BD could be affected by other factors, such as life stress. Further study on the correlation between distress and disorder could be conducted.

In conclusion, the early detection of a potential endo-phenotype of bipolar spectrum disorder is important due to the clinical implications, and may be used in monitoring the progression of the disorder (59). Thus far, no effect of subtypes on EI performance was noted; however, the disorder course had a greater impact instead. EI may be used as a measurement, and the subscales could be used as a profile to compare different mental disorders and could be a marker of disorder progression. More studies with longitudinal follow-up are needed to investigate the stability of EI and its association with the course of BD. A further intervention regarding coping skills based on the course of BD could then be possible.

## Data Availability Statement

The raw data supporting the conclusions of this article will be made available by the authors, without undue reservation.

## Ethics Statement

The studies involving human participants were reviewed and approved by Institute Review Board (IRB) of the National Cheng Kung University Hospital and the China Medical University Hospital. The patients/participants provided their written informed consent to participate in this study.

## Author Contributions

S-YK wrote the first draft of this manuscript with Y-HC. T-YW, C-CH, H-HT, PSC, YKY, H-YL, and R-BL managed patient recruitment and procedure of this study. S-YK, Y-HC, and PSC were in respondent to data analyses. All authors have read and had approval of this manuscript.

## Conflict of Interest

The authors declare that the research was conducted in the absence of any commercial or financial relationships that could be construed as a potential conflict of interest.

## References

[1] Hsu PC Chen HC Lu MJ Lu RB Lin CE. Care of individuals with bipolar disorders. Hu Li Za Zhi. (2017) 64:19–26. 10.6224/JN.000036 28580555

[2] Summers M Papadopoulou K Bruno S Cipolotti L Ron MA. Bipolar I and bipolar II disorder: cognition and emotion processing. Psychol Med. (2006) 36:1799–809. 10.1017/S0033291706008804 16938147

[3] Zhang Z Lindpaintner K Che R He Z Wang P Yang P. The Val/Met functional polymorphism in COMT confers susceptibility to bipolar disorder: evidence from an association study and a meta-analysis. J Neural Transm (Vienna). (2009) 116:1193–200. 10.1007/s00702-009-0260-7 19578924

[4] Goldberg J. Consistency of remission and outcome in bipolar and unipolar mood disorders: a 10-year prospective follow-up^*^1. J Affect Disord. (2004) 81:123–31. 10.1016/s0165-0327(03)00161-7 15306137

[5] Craddock N Sklar P. Genetics of bipolar disorder. Lancet. (2013) 381:1654–62. 10.1016/s0140-6736(13)60855-7 23663951

[6] Rosa AR Reinares M Franco C Comes M Torrent C Sánchez-Moreno J. Clinical predictors of functional outcome of bipolar patients in remission. Bipolar Disord. (2009) 11:401–9 19500093 10.1111/j.1399-5618.2009.00698.x

[7] Samame C. Social cognition throughout the three phases of bipolar disorder: a state-of-the-art overview. Psychiatry Res. (2013) 210:1275–86. 10.1016/j.psychres.2013.08.012 24075306

[8] Townsend J Altshuler LL. Emotion processing and regulation in bipolar disorder: a review. Bipolar Disord. (2012) 14:326–39. 10.1111/j.1399-5618.2012.01021.x 22631618

[9] Burdick KE Goldberg JF Harrow M. Neurocognitive dysfunction and psychosocial outcome in patients with bipolar I disorder at 15-year follow-up. Acta Psychiatr Scand. (2010) 122:499–506. 10.1111/j.1600-0447.2010.01590.x 20637012 PMC2980854

[10] Burdick KE Goldberg TE Cornblatt BA Keefe RS Gopin CB DeRosse P. The MATRICS consensus cognitive battery in patients with bipolar I disorder. Neuropsychopharmacology. (2011) 36:1587–92. 10.1038/npp.2011.36 21451499 PMC3138672

[11] Getz GE Shear PK Strakowski SM. Facial affect recognition deficits in bipolar disorder. J Int Neuropsychol Soc. (2003) 9:623–32. 10.1017/S1355617703940021 12755174

[12] Venn HR Gray JM Montagne B Murray LK Michael Burt D Frigerio E. Perception of facial expressions of emotion in bipolar disorder. Bipolar Disord. (2004) 6:286–93. 10.1111/j.1399-5618.2004.00121.x 15225145

[13] Gruber J Hay AC Gross JJ. Rethinking emotion: cognitive reappraisal is an effective positive and negative emotion regulation strategy in bipolar disorder. Emotion. (2014) 14:388–96. 10.1037/a0035249 24364852

[14] Samame C Martino DJ Strejilevich SA. An individual task meta-analysis of social cognition in euthymic bipolar disorders. J Affect Disord. (2015) 173:146–53. 10.1016/j.jad.2014.10.055 25462409

[15] Amodio DM Frith CD. Meeting of minds: the medial frontal cortex and social cognition. Nat Rev Neurosci. (2006) 7:268–77. 10.1038/nrn1884 16552413

[16] Lee J Altshuler L Glahn DC Miklowitz DJ Ochsner K Green MF. Social and nonsocial cognition in bipolar disorder and schizophrenia: relative levels of impairment. Am J Psychiatry. (2013) 170:334–41. 10.1176/appi.ajp.2012.12040490 23450289 PMC3869864

[17] Mao WC Chen LF Chi CH Lin CH Kao YC Hsu WY. Traditional Chinese version of the Mayer Salovey Caruso Emotional Intelligence Test (MSCEIT-TC): its validation and application to schizophrenic individuals. Psychiatry Res. (2016) 243:61–70. 10.1016/j.psychres.2016.04.107 27367492

[18] Yatham LN Torres IJ Malhi GS Frangou S Glahn DC Bearden CE. The international society for bipolar disorders-battery for assessment of neurocognition (ISBD-BANC). Bipolar Disord. (2010) 12:351–63. 10.1111/j.1399-5618.2010.00830.x 20636632

[19] Beer JS Ochsner KN. Social cognition: a multi level analysis. Brain Res. (2006) 1079:98–105. 10.1016/j.brainres.2006.01.002 16513097

[20] Green MF Penn DL Bentall R Carpenter WT Gaebel W Gur RC. Social cognition in schizophrenia: an NIMH workshop on definitions, assessment, and research opportunities. Schizophr Bull. (2008) 34:1211–20. 10.1093/schbul/sbm145 18184635 PMC2632490

[21] Mancuso F Horan WP Kern RS Green MF. Social cognition in psychosis: multidimensional structure, clinical correlates, and relationship with functional outcome. Schizophr Res. (2011) 125:143–51. 10.1016/j.schres.2010.11.007 21112743 PMC3073542

[22] Mayer J. MSCEIT: Mayer-Salovey-Caruso Emotional Intelligence Test. Toronto, Canada: Multi-Health Systems (2002).

[23] Ochsner KN. The social-emotional processing stream: five core constructs and their translational potential for schizophrenia and beyond. Biol Psychiatry. (2008) 64:48–61. 10.1016/j.biopsych.2008.04.024 18549876 PMC2453243

[24] Soeiro-de-Souza MG Bio DS David DP Rodrigues dos Santos D Jr Kerr DS Gattaz WF. COMT Met (158) modulates facial emotion recognition in bipolar I disorder mood episodes. J Affect Disord. (2012) 136:370–6. 10.1016/j.jad.2011.11.021 22222175

[25] Salovey P Mayer JD. Emotional intelligence. Imagin, Cogn Pers. (1990) 9:185–211.

[26] Mayer JD Salovey P Caruso DR Sitarenios G. Measuring emotional intelligence with the MSCEIT V2.0. Emotion. (2003) 3:97–105. 10.1037/1528-3542.3.1.97 12899321

[27] Mayer JD Salovey P Caruso DR. Emotional intelligence: new ability or eclectic traits? Am Psychol. (2008) 63:503–17. 10.1037/0003-066X.63.6.503 18793038

[28] Mayer JD Caruso DR Salovey P. Emotional intelligence meets traditional standards for an intelligence. Intelligence. (1999) 27:267–98.12934682

[29] Mayer JD Geher G. Emotional intelligence and the identification of emotion. Intelligence. (1996) 22:89–113. 10.1016/S0160-2896(96)90011-2

[30] Tabak NT Green MF Wynn JK Proudfit GH Altshuler L Horan WP. Perceived emotional intelligence is impaired and associated with poor community functioning in schizophrenia and bipolar disorder. Schizophr Res. (2015) 162:189–95. 10.1016/j.schres.2014.12.005 25579055 PMC4339495

[31] Kee KS Horan WP Salovey P Kern RS Sergi MJ Fiske AP. Emotional intelligence in schizophrenia. Schizophr Res. (2009) 107:61–8. 10.1016/j.schres.2008.08.016 18805674

[32] Smieja M Orzechowski J Stolarski MS. TIE: an ability test of emotional intelligence. PLoS ONE. (2014) 9:e103484. 10.1371/journal.pone.0103484 25072656 PMC4114749

[33] Aparicio A Santos JL Jimenez-Lopez E Bagney A Rodriguez-Jimenez R Sanchez-Morla EM. Emotion processing and psychosocial functioning in euthymic bipolar disorder. Acta Psychiatr Scand. (2017) 135:339–50. 10.1111/acps.12706 28188631

[34] Varo C Jimenez E Sole B Bonnin CM Torrent C Lahera G. Social cognition in bipolar disorder: the role of sociodemographic, clinical, and neurocognitive variables in emotional intelligence. Acta Psychiatr Scand. (2019) 139:369–80. 10.1111/acps.13014 30786002

[35] Varo C Jimenez E Sole B Bonnin CM Torrent C Valls E. Social cognition in bipolar disorder: focus on emotional intelligence. J Affect Disord. (2017) 217:210–17. 10.1016/j.jad.2017.04.012 28427032

[36] Bo Q Mao Z Li X Wang Z Wang C Ma X. Use of the MATRICS consensus cognitive battery (MCCB) to evaluate cognitive deficits in bipolar disorder: a systematic review and meta-analysis. PLoS ONE. (2017) 12:e0176212. 10.1371/journal.pone.0176212 28437438 PMC5402962

[37] Burdick KE Russo M Frangou S Mahon K Braga RJ Shanahan M. Empirical evidence for discrete neurocognitive subgroups in bipolar disorder: clinical implications. Psychol Med. (2014) 44:3083–96. 10.1017/S0033291714000439 25065409 PMC4797987

[38] Fan H Jackson T Yang X Tang W Zhang J. The factor structure of the Mayer–Salovey–caruso emotional intelligence test V 2.0 (MSCEIT): a meta-analytic structural equation modeling approach. Pers Individ Differ. (2010) 48:781–5. 10.1016/j.paid.2010.02.004

[39] Gardner KJ Qualter P. Factor structure, measurement invariance and structural invariance of the MSCEIT V2.0. Pers Individ Differ. (2011) 51:492–6. 10.1016/j.paid.2011.05.004

[40] Keefe RS Fox KH Harvey PD Cucchiaro J Siu C Loebel A. Characteristics of the MATRICS consensus cognitive battery in a 29-site antipsychotic schizophrenia clinical trial. Schizophr Res. (2010) 125:161–8. 10.1016/j.schres.2010.09.015 21075600

[41] Kern RS Nuechterlein KH Green MF Baade LE Fenton WS Gold JM. The MATRICS consensus cognitive battery, part 2: co-norming and standardization. Am J Psychiatry. (2008) 165:214–20. 10.1176/appi.ajp.2007.07010043 18172018

[42] Martino DJ Strejilevich SA Fassi G Marengo E Igoa A. Theory of mind and facial emotion recognition in euthymic bipolar I and bipolar II disorders. Psychiatry Res. (2011) 189:379–84. 10.1016/j.psychres.2011.04.033 21620484

[43] Vaskinn A Lagerberg TV Bjella TD Simonsen C Andreassen OA Ueland T. Impairment in emotion perception from body movements in individuals with bipolar I and bipolar II disorder is associated with functional capacity. Int J Bipolar Disord. (2017) 5:13. 10.1186/s40345-017-0083-7 28332121 PMC5433954

[44] Van Rheenen TE Rossell SL. Objective and subjective psychosocial functioning in bipolar disorder: an investigation of the relative importance of neurocognition, social cognition and emotion regulation. J Affect Disord. (2014) 162:134–41. 10.1016/j.jad.2014.03.043 24767018

[45] Tohen M Zarate Jr CA Hennen J Khalsa H-MK Strakowski SM Gebre-Medhin P. The McLean-Harvard first-episode mania study: prediction of recovery and first recurrence. Am J Psychiatry. (2003) 160:2099–107. 10.1176/appi.ajp.160.12.2099 14638578

[46] Gutierrez-Rojas L Jurado D Gurpegui M. Factors associated with work, social life and family life disability in bipolar disorder patients. Psychiatry Res. (2011) 186:254–60. 10.1016/j.psychres.2010.06.020 20647154

[47] Bearden CE Shih VH Green MF Gitlin M Sokolski KN Levander E. The impact of neurocognitive impairment on occupational recovery of clinically stable patients with bipolar disorder: a prospective study. Bipolar Disord. (2011) 13:323–33. 10.1111/j.1399-5618.2011.00928.x 21843272 PMC3157039

[48] Bonnin CM Torrent C Goikolea JM Reinares M Sole B Valenti M. The impact of repeated manic episodes and executive dysfunction on work adjustment in bipolar disorder. Eur Arch Psychiatry Clin Neurosci. (2014) 264:247–54. 10.1007/s00406-013-0431-2 23912643

[49] Martino DJ Marengo E Igoa A Scapola M Ais ED Perinot L. Neurocognitive and symptomatic predictors of functional outcome in bipolar disorders: a prospective 1 year follow-up study. J Affect Disord. (2009) 116:37–42. 10.1016/j.jad.2008.10.023 19033081

[50] Liu YC Tseng H-H Chang Y-H Chang HH Yang YK Chen PS. The social cognitive ability in Han Chinese euthymic patients with bipolar I and bipolar II disorder. Journal of the Formosan Medical Association. (2020). 10.1016/j.jfma.2020.10.012 34756401

[51] Huang CC Chang YH. Effects of mood episodes and comorbid anxiety on neuropsychological impairment in patients with bipolar spectrum disorder. Brain Behav. (2020) 10:e01813. 10.1002/brb3.1813 32864897 PMC7667309

[52] Young RC Biggs JT Ziegler VE Meyer DA. A rating scale for mania: reliability, validity and sensitivity. Br J Psychiatry. (1978) 133:429–35. 10.1192/bjp.133.5.429 728692

[53] Hamilton M. Development of a rating scale for primary depressive illness. Br J Soc Clin Psychol. (1967) 6:278–96. 6080235 10.1111/j.2044-8260.1967.tb00530.x

[54] Hamilton M. A rating scale for depression. J Neurol Neurosurg Psychiatry. (1960) 23:56–62. 14399272 10.1136/jnnp.23.1.56 PMC495331

[55] Furukawa TA. Assessment of mood: guides for clinicians. J Psychosom Res. (2010) 68:581–9. 10.1016/j.jpsychores.2009.05.003 20488276

[56] Ma WF Tsai GE Chang JP Lane HY. Reliability and validity of three Chinese-version tasks of Mayer-Salovey-Caruso Emotional Intelligence Test. J Clin Nurs. (2010) 19:2656–8. 10.1111/j.1365-2702.2010.03316.x 20920087

[57] Cabello R Navarro Bravo B Latorre JM Fernández-Berrocal P. Ability of university-level education to prevent age-related decline in emotional intelligence. Front Aging Neurosci. (2014) 6:37. 10.3389/fnagi.2014.00037 24653697 PMC3949193

[58] Martins FMP Leite KP Trevizol AP Noto JRS Brietzke E. Emotional intelligence and schizophrenia spectrum disorders: a critical review. Trends Psychiatry Psychother. (2019) 41:94–102. 10.1590/2237-6089-2018-0001 30994788

[59] Frajo-Apor B Kemmler G Pardeller S Huber M Macina C Welte A-S. Emotional intelligence in bipolar-I-disorder: a comparison between patients, unaffected siblings, and control subjects. Eur Psychiatry. (2020) 63:e69. 10.1192/j.eurpsy.2020.66 32594936 PMC7443786

[60] Cabello R Sorrel M Fernández-Pinto I Extremera N Fernández-Berrocal P. Age and gender differences in ability emotional intelligence in adults: a cross-sectional study. Dev Psychol. (2016) 52:1486–92. 10.1037/dev0000191 27570984

[61] Lahera G Herrera S Reinares M Benito A Rullas M Gonzalez-Cases J. Hostile attributions in bipolar disorder and schizophrenia contribute to poor social functioning. Acta Psychiatr Scand. (2015) 131:472–82. 10.1111/acps.12399 25645449

[62] Judd LL Akiskal HS Schettler PJ Endicott J Leon AC Solomon DA. Psychosocial disability in the course of bipolar I and II disorders: a prospective, comparative, longitudinal study. Arch Gen Psychiatry. (2005) 62:1322–30. 10.1001/archpsyc.62.12.1322 16330720

[63] Dodd A Lockwood E Mansell W Palmier-Claus J. Emotion regulation strategies in bipolar disorder: A systematic and critical review. J Affect Disord. (2019) 246:262–84. 10.1016/j.jad.2018.12.026 30590289

[64] Martinez-Aran A Vieta E Reinares M Colom F Torrent C Sanchez-Moreno J. Cognitive function across manic or hypomanic, depressed, and euthymic states in bipolar disorder. Am J Psychiatry. (2004) 161:262–70. 10.1176/appi.ajp.161.2.262 14754775

